# Special Issue “Plant Viruses: From Ecology to Control”

**DOI:** 10.3390/microorganisms9061136

**Published:** 2021-05-25

**Authors:** Jesús Navas-Castillo, Elvira Fiallo-Olivé

**Affiliations:** Instituto de Hortofruticultura Subtropical y Mediterránea “La Mayora”, Consejo Superior de Investigaciones Científicas, Universidad de Málaga (IHSM-CSIC-UMA), Avenida Dr. Wienberg s/n, 29750 Algarrobo-Costa, Málaga, Spain; efiallo@eelm.csic.es

Plant viruses cause many of the most important diseases threatening crops worldwide. Over the last quarter of a century, an increasing number of plant viruses have emerged in various parts of the world, especially in the tropics and subtropics. As is generally observed for plant viruses, most of the emerging viruses are transmitted horizontally by biological vectors, mainly insects. Reverse genetics using infectious clones—available for many plant viruses—have been used for the identification of viral determinants involved in virus–host and virus–vector interactions. Although many studies have identified a number of factors involved in disease development and transmission, the precise mechanisms are unknown for most of the virus–plant–vector combinations. In most cases, the diverse outcomes resulting from virus–virus interactions are poorly understood. Although significant advances have been made towards understanding the mechanisms involved in plant resistance to viruses, we are far from being able to apply this knowledge to protect cultivated plants from all viral threats.

The aim of this Special Issue was to provide a platform for researchers interested in plant viruses to share their recent results related to the various aspects of plant virology: ecology, virus–plant host interactions, virus–vector interactions, virus–virus interactions, and control strategies. A total of 15 papers have been contributed by 96 authors from 18 countries to the issue, comprising ten research articles, one short communication, and four reviews (Figure 1).

Plant virus ecology looks at virus–host-environment interactions and, in a broad sense, includes studies on biodiversity and evolution. Several papers in this Special Issue focused on this topic. Martínez-Turiño et al. [1] described how virus host jumping, from *Nicotiana clevelandii* to *Chenopodium foetidum*, can be boosted by adaptation to a bridge plant species, *Arabidopsis thaliana*, using mutants of the potyvirus plum pox virus. Kim et al. [2] also analyzed the host adaptation process by characterizing field isolates of the capillovirus apple stem grooving virus sampled from two plant hosts, apple and pear trees, revealing that host adaptation was influenced by the host’s codon-usage. Ferro et al. [3] studied the complexity of sweepovirus-deltasatellite-plant host interactions by looking at the diversity of satellite and helper virus natural populations, as well as by performing co-inoculation experiments to assess the ability of a number of geminivirids to transreplicate sweet potato leaf curl deltasatellite 1. Montes et al. [4], using a genome-wide association strategy, identified a number of genes associated with virulence in *Arabidopsis thaliana* genotypes infected by the cucumovirus cucumber mosaic virus that were linked to virus seed transmission. On a more applied aspect, Nancarrow et al. [5] estimated the yield losses caused by the luteovirus barley yellow dwarf virus-PAV in wheat and barley by conducting a three-year field study in Australia, highlighting the importance of performing this type of study under varying conditions for specific cultivar–vector–virus combinations.

High-throughput sequencing (HTS) technologies have become indispensable tools to characterize plant virus diversity thanks to their ability to detect virtually any virus without prior sequence knowledge. Three papers published in this Special Issue deal with HTS. Kutnjak et al. [6] present a critical overview, useful for both beginners and expert scientists interested on plant virome projects, of the steps involved in HTS, including available bioinformatic tools and the steps to be used to implement a structured data analysis. Maachi et al. [7] used HTS to identify the viruses present in a number of symptomatic tomato plant samples using two RNA purification methods, illustrating its usefulness with the first identification of three viruses infecting this crop in Spain. Ben Chehida et al. [8] went further by showing that nanopore sequencing is a reliable alternative for obtaining complete genome sequences of geminivirids, which is also applicable to other circular ssDNA viruses.

The study of the precise interactions established between viruses and plant hosts to cause a productive infection is a fundamental subject that has received the attention of three research groups contributing to this Special Issue. Teixeira et al. [9] reviewed the different levels of geminivirid perception and defense mechanisms acting in the plant host, as well as the counter-defense strategies evolved by geminivirids to overcome such mechanisms of resistance. Kumar and Dasgupta [10] reviewed another interesting topic within the scope of this Special Issue, the movement proteins encoded by virus genomes, focusing on structural diversity, multifunctionality, and interaction with plant host proteins. Leastro et al. [11] analyzed the association of the movement and capsid proteins of the cilevirus citrus leprosis virus C2 with plant cell membranes by using computer predictions and molecular assays, discussing the implications of their findings for the intracellular movement of the virus.

Deciphering virus–vector interactions is an emerging research subject in plant virology. Thus far, studies concerning begomoviruses, an important group of emerging pathogens transmitted by the whiteflies of the *Bemisia tabaci* complex, have been mostly conducted with tomato yellow leaf curl virus. In this Special Issue, Chi et al. [12] showed that a vacuolar protein sorting-associated protein from the Asia II 1 whitefly interacts with the coat protein of the begomovirus cotton leaf curl Multan virus, suggesting that this protein may play an important role in transmission.

Mixed-infections with two or more plant viruses are frequent in the field, with viruses being able to interact in multiple and intricate ways. These interactions are generally categorized as synergistic, antagonistic, or neutral. In this Special Issue, Elvira González et al. [13] experimentally analyzed the interactions between an asymptomatic persistent virus, the amalgavirus southern tomato virus, and two well-known acute viruses infecting tomato, the cucumovirus cucumber mosaic virus and the potexvirus pepino mosaic virus, showing that the persistent virus caused a synergistic effect.

Although a number of biotechnological approaches have been developed to produce virus-resistant plants in recent years, the use of classical genetic resistance remains the strategy of choice for practical control of plant viruses. In this Special Issue, Yan et al. [14] reviewed the genetic resources used in breeding for resistance to one of the most harmful viral disease affecting tomato globally, tomato yellow leaf curl disease and the begomoviruses that cause it. The authors also summarized some of the future studies aimed at increasing the success and durability of genetic resistance to these and related viruses in a scenario of globalization, climate change, and viral disease emergence. Finally, Sáez et al. [15] reported the search for resistance to a cucurbit-adapted strain of tomato leaf curl New Delhi virus, a severe threat for cucurbit production worldwide, in cucumber. They identified, by the first-time, cucumber accessions highly resistant to the virus, and were successful in characterizing the mode of inheritance and location in the cucumber genome of the resistance gene.

Overall, the papers in this Special Issue reveal different perspectives of current research on plant viruses, from applied field studies to investigations into the intricate mechanisms involved in the tripartite interactions between viruses, plants, and vectors.

## Figures and Tables

**Figure 1:**
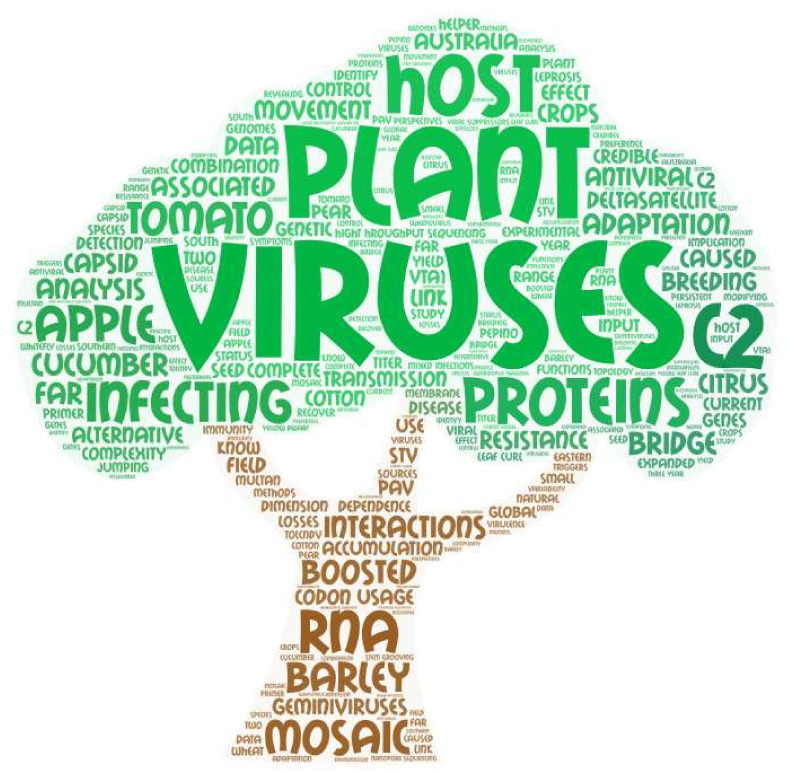
A word cloud created from the titles of every article published in this Special Issue.
